# The protein tyrosine phosphatase PPH‐7 is required for fertility and embryonic development in *C. elegans* at elevated temperatures

**DOI:** 10.1002/2211-5463.13771

**Published:** 2024-02-06

**Authors:** Curdin A. Franziscus, Danilo Ritz, N. Constantin Kappel, Jachen A. Solinger, Alexander Schmidt, Anne Spang

**Affiliations:** ^1^ Biozentrum University of Basel Switzerland; ^2^ Leica Microsystems CMS GmbH Wetzlar Germany

**Keywords:** *C. elegans*, phosphatase, phosphorylation, post‐translational modifications, protein tyrosine phosphatase, PTPN

## Abstract

Post‐translational modifications are key in the regulation of activity, structure, localization, and stability of most proteins in eukaryotes. Phosphorylation is potentially the most studied post‐translational modification, also due to its reversibility and thereby the regulatory role this modification often plays. While most research attention was focused on kinases in the past, phosphatases remain understudied, most probably because the addition and presence of the modification is more easily studied than its removal and absence. Here, we report the identification of an uncharacterized protein tyrosine phosphatase PPH‐7 in *C. elegans*, a member of the evolutionary conserved PTPN family of phosphatases. Lack of PPH‐7 function led to reduction of fertility and embryonic lethality at elevated temperatures. Proteomics revealed changes in the regulation of targets of the von Hippel–Lindau (VHL) E3 ligase, suggesting a potential role for PPH‐7 in the regulation of VHL.

## Graphical abstract

Using long‐read sequencing, we identify the genetic lesion in a temperature‐sensitive *C. elegans* mutant. We find that a previously uncharacterized tyrosine phosphatase (PPH‐7) is required for spermatogenesis and embryonic development at elevated temperatures. Analysis of the proteome revealed that multiple known targets of the Von Hippel–Lindau tumor suppressor are downregulated in PPH‐7 deficient mutants.

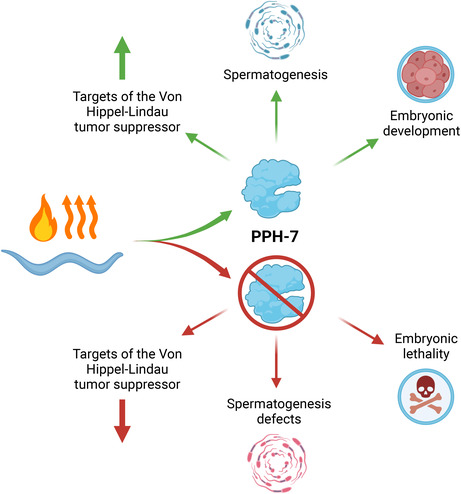

**Abbreviations**
CC1: cysteine‐based class I
DSP: dual specificity phosphatase
ECM: extracellular matrix
EMS: ethyl methane sulfonate
FDR: false discovery rate
HCD: higher‐energy collisional dissociation
HIF: hypoxia‐inducible factor
MIPS: monoisotopic precursor selection
MSP: major sperm protein
PTEN: phosphatase and tensin homolog
PTK: protein tyrosine kinase
PTP: protein tyrosine phosphatase
PTPN: protein tyrosine phosphatase non‐receptor type
PTPR: protein tyrosine phosphatase receptor type
SNP: single nucleotide polymorphism
VHL: Von Hippel–Lindau
wt: wild type

Protein phosphorylation is a reversible post‐translational modification that plays a crucial role in the regulation of protein structure, activity, and function *in vivo*. While most of the protein phosphorylation occurs on serine and threonine residues, tyrosine phosphorylation only accounts for 1.8% of the phospho‐amino acids found in cells [1]. However, numerous human diseases are associated with dysregulation of protein tyrosine phosphorylation including multiple types of cancer [2, 3, 4, 5], highlighting the importance of an intact balance between phosphorylation and dephosphorylation of tyrosine residues. This balance is ensured by an interplay between protein tyrosine kinases (PTKs) and protein tyrosine phosphatases (PTPs), adding and removing phosphate groups on tyrosine residues, respectively. Historically, protein kinases have been studied more in‐depth than their phosphatase counterparts. However, over the past years, it has become clear that better understanding of phosphatases and their role in the regulation of cellular processes is necessary for an integrated understanding of these processes and the diseases associated with their dysregulation [6, 7, 8, 9].

Humans express 81 active PTPs and 85 active PTKs [10]. This complexity is only mildly reduced in the nematode *C. elegans*, which expresses 57 PTPs, most of which remain unstudied [11]. These 57 active PTPs are outnumbered by 92 active PTKs [12], indicating that the phosphatases exhibit a broader substrate specificity than their kinase counterparts. *C. elegans* with its short generation time and simple body plan provides an excellent tool to analyze the function of phosphatases and kinases on an organismal level.

Mutations in the Von Hippel–Lindau tumor suppressor (VHL) gene in humans have been associated with multiple types of cancer [13]. The most established function of the E3 ubiquitin ligase VHL is the regulation of the levels of the transcription factor Hypoxia‐Inducible Factor (HIF) by targeting it for proteasomal degradation under normoxic conditions [14, 15, 16, 17]. Under hypoxic conditions or in the absence of VHL, HIF is stabilized and HIF target genes, which are frequently associated with cancer, such as TGF‐α, are overexpressed [18]. In *C. elegans*, HIF‐1 is supposedly required for numerous stress responses, including acclimatization to heat stress and thermotolerance [19, 20]. In addition, VHL has other targets that are independent of HIF‐1 in *C. elegans*, mice and cancer cells [21, 22, 23]. Those VHL targets have been often connected to the extracellular matrix (ECM) in *C. elegans* and mice [21, 24].

In this study, we used a forward genetics approach to identify the genetic lesion in a mutant (FA50) that exhibits both a severe reduction in fertility and an increase in embryonic lethality at the elevated temperature of 26 °C. Using long‐read sequencing, we located the mutation to the gene encoding the uncharacterized protein tyrosine phosphatase PPH‐7. This phosphatase exhibits homology to the human protein tyrosine phosphatase non‐receptor type (PTPN) gene family, which plays a role in a plethora of cellular processes. Proteomics and phosphoproteomics of *pph‐7* mutant animals at restrictive temperature revealed a strong downregulation of known *vhl‐1* targets, suggesting a role of PPH‐7 in the Von Hippel–Lindau tumor suppressor pathway.

## Materials and methods

### Worm husbandry

The *C. elegans* worms were crossed and maintained using standard methods [25]. All strains were kept at 15 °C unless mentioned otherwise.

The following worm strains were used in this study: N2, CB4865, FA50: *pph‐7*(*af5*), FA54: *pph‐7*(*af5*) x CB4856, tm5332: *pph‐7*(*tm5332*).

### SNP mapping of mutation in FA50

Ten males of the mapping strain CB4856 (which carries numerous known SNPs compared to N2) were crossed to 5 mutant hermaphrodites generated in an N2 background. The F1 worms (which are heterozygous for the mutation) were moved to new plates to avoid starvation. 400–1000 F2 worms (1/4 are homozygous for mutation) were singled out onto individual plates. In the F3 generation 10 worms, for every F2 worm which was singled out, were put onto a new plate and shifted to 26 °C. The plates which showed little to no offspring were considered homozygous for the mutation and the corresponding plates at 15 °C were used for DNA isolation. Equal amounts of plates producing offspring (heterozygous or WT plates) were also used for DNA extraction. The isolated DNA of both samples was then used as a template to perform a PCR reaction amplifying the region surrounding the SNP of interest. Subsequently, the PCR products were digested using restriction enzymes which only cut either the N2 sequence or the sequence found in CB4856. The resulting digested PCR products were used for gel electrophoresis. Based on the observed band intensity for the distinct bands, the map ration was calculated as follows: Map ratio = (Mutant_N2 band_/Mutant_CB band_)/(WT_N2 band_/WT_CB band_). Hence, a low map ratio is an indication that the corresponding SNP is in proximity of the mutation. The map ratio was determined for 3 SNPs spreading across the whole chromosome length on every chromosome (exact position of SNPs is shown in Table S1). Method adapted from Wicks *et al*. [26].

### Imaging of temperature‐sensitive phenotypes

16 L1 larvae were moved to a new 60 mm NGM plate and incubated at 26 °C for 5 days. After 5 days the plates were imaged using a PowerShot G16 Digital Camera (Canon, Tokyo, Japan) mounted on a Zeiss Stemi 2000‐C Stereo Microscope (ZEISS, Jena, Germany).

### *C. elegans* liquid culture for genomic DNA

*Escherichia coli* OP50 was grown in 1 L of LB media to an OD_600_ of 3, spun down and resuspended in 1% of the culture volume in S medium [27] (final OD ~ 300). This solution serves as food solution for the worms. One 60 mm NGM plate full of worms was washed off and the worms were added to 100 mL of S medium (+100 U·mL^−1^ Nystatin) together with 1 mL of food solution. The liquid culture was incubated at 20 °C, 200 rpm and 1 mL of food solution was added every time the culture started becoming clear. As soon as the culture reached ~ 10 worms·μL^−1^ (6–8 days) the culture was spun down, and the worms were washed three times with S medium. Finally, the worm pellet was aliquoted into 5–10 pellets each weighing 100 mg, flash frozen in liquid nitrogen and stored at −80 °C until DNA extraction.

### Extraction of long DNA for deep sequencing

DNA extraction was performed from flash frozen 100 mg worm pellets using a non‐publicly available beta version of the Nanobind Tissue Big DNA Kit (Pacific Biosciences, Menlo Park, CA, USA) with optimized buffers for *C. elegans* (contact Pacific Biosciences directly for more information). This resulted in 10–15 μg of DNA per sample with an average fragment length of 43–58 kb.

### PacBio sequencing

After an AMPure purification step and cutting of the fragment to 10 kb size using the BluePippin system, an SMRTbell library was prepared. Subsequent sequencing on the SMRT cell yielded between 197 000 and 325 000 circular consensus sequence HiFi reads (Q20 quality filtered) with a mean length of 8000–10 000 base pairs for each of the three analyzed strains. This results in 1 900 000 000‐2 700 000 000 sequenced base pairs per strain. 100% of these reads had a base calling accuracy of > 99% (can be used for alignment), while 80% of the reads had a base calling accuracy of > 99.9%.

### Alignment of sequencing reads

As a reference genome the N2 genome assembly WBcel235 (WormBase release WS283) was used. The sequencing reads were aligned using the built‐in Map Reads to Reference tool of the clc genomic workbench (22.0.2) software (Qiagen, Venlo, Netherlands) (Parameters: References = *Caenorhabditis elegans* (WBcel235), Masking mode = No masking, Match score = 1, Mismatch cost = 2, Cost of insertions and deletions = Linear gap cost, Insertion cost = 3, Deletion cost = 3, Length fraction = 0.5, Similarity fraction = 0.8, Global alignment = No, Non‐specific match handling = Map randomly, Output mode = Create reads track, Create report = No, Collect unmapped reads = No). Between 181 000 and 246 000 reads were successfully mapped for each analyzed strain resulting in an average coverage between 16‐ and 22‐fold, which is sufficient for accurate variant calling and assembly [28].

### Sequencing data analysis

The sequencing data were analyzed using the clc genomic workbench (22.0.2) software. The built‐in Basic Variant Detection tool was used to detect variants in all three analyzed strains (Parameters: Ploidy = 2, Ignore positions with coverage above = 100 000, Restrict calling to target regions = Not set, Ignore broken pairs = Yes, Ignore non‐specific matches = Reads, Minimum coverage = 4, Minimum count = 2, Minimum frequency (%) = 35.0, Base quality filter = No, Read direction filter = No, Relative read direction filter = Yes, Significance (%) = 1.0, Read position filter = No, Remove pyro‐error variants = No, Create track = Yes, Create annotated table = No). Subsequently, the variants found in the mutant strains were filtered for their zygosity and only homozygous variants were included for further analysis. Next, all variants found in the wild‐type control were subtracted from the homozygous variants found in both mutant strains using the Filter against Known Variants tool (Parameters: Join adjacent MNVs and SNVs = No, Filter action = Keep variants with no exact match found in the track of known variants). Again, using the Filter against Known Variants tool, the remaining variants that are shared between both mutant strains were determined (Parameters: Join adjacent MNVs and SNVs = No, Filter action = Keep variants with exact match found in the track of known variants). Finally, using the Amino Acid Changes tool the variants that cause a change in the amino acid sequence were identified (Parameters: CDS track = *Caenorhabditis elegans* (WBcel235.107)_CDS, Sequence track = *Caenorhabditis elegans* (WBcel235), mRNA track = *Caenorhabditis elegans* (WBcel235.107)_mRNA, Use transcript priorities = No, Move variants from VCF location to HGVS location = No, Include upstream flanking positions = 5000, Include downstream flanking positions = 2000, Filter away synonymous variants = Yes, Genetic code = 1 Standard, Filter away CDS regions with no variants = Yes).

### Diagnostic PCR

Diagnostic PCR was used to follow the 553 bp deletion in *tm5332* as well as the 2‐bp deletion in *af5* during crosses. 5–10 worms were picked into a PCR tube containing 1× PCR buffer and 0.5 mg·mL^−1^ proteinase K. Next, worms were flash frozen at and stored at −80 °C until further use. Lysis was done by heating samples to 65 °C for 60 min, followed by heat inactivation of proteinase K at 95 °C for 15 min in the thermocycler. Finally, PCR master mix containing the corresponding primers was added and the PCR was started. Primers used to follow 553 bp deletion in *tm5332*: fwd 5′‐GAATATGGAGAACACTCCG‐3′, rev (wild type) 5′‐GAAATGTACTTGGCGCAC‐3′ and rev (mutant) 5′‐TGACAAATCCGACGATGTC‐3′. Primers used to follow 2 bp deletion in *af5*: fwd 5′‐AAGATTGGTCCTGCAAATAC‐3′ and rev 5′‐AGTGGATCCCAGAGACAATG‐3′. Gel electrophoresis was performed on the PCR product. For the 553 bp deletion in *tm5332*, the length difference of the PCR products between mutants and wild type was sufficient for genotyping, while for the 2 bp deletion in *af5* the resulting PCR product was sent for Sanger sequencing at Microsynth AG (Balgach, Switzerland).

### Cloning of rescue plasmid

The Y71F9AL.4/*pph‐7* promotor was amplified from *C. elegans* gDNA using PCR and cloned into the pPD95.79 vector (Addgene, Watertown, MA, USA), which was previously cut by *Xba*I and *Kpn*I, using the NEBuilder HiFi DNA Assembly Cloning Kit (New England BioLabs, Ipswich, MA, USA) and the following primers. Primers for Y71F9AL.4/*pph‐7* promotor: fwd 5′‐tgcatgcctgcaggtcgactctagaGATAATCAGCCAGATGCTC‐3′ and rev 5′‐ctcattttttctaccggtaccTTTACTAGAATCCCAATTGATTTTTTTG‐3′. In a second step, the Y71F9AL.4/*pph‐7* gene was amplified from *C. elegans* genomic DNA using PCR and cloned into the pPD95.79 vector after the already inserted Y71F9AL.4/*pph‐7* promotor again using the NEBuilder HiFi DNA Assembly Cloning Kit (New England BioLabs). Primers for Y71F9AL.4/*pph‐7*: fwd 5′‐ggtagaaaaaATGTATCACAAGACAGGTAAG‐3′ and rev 5′‐tctacgaatgCTATTCACGCTTCTCAATG‐3′. Primers to linearize vector: fwd 5′‐gcgtgaatagCATTCGTAGAATTCCAACTG‐3′ and rev 5′‐tgtgatacatTTTTTCTACCGGTACCTTTAC‐3′. The resulting plasmid was confirmed using Sanger sequencing performed by Microsynth AG.

### Microinjection of rescue plasmids

One droplet of halocarbon oil was pipetted onto the injection pad (dried 2% agarose on a glass slide). Worms were picked into the oil droplet and gently pushed down so that they stick to the pad and are immobilized. Subsequently, the injection mix [20 ng·μL^−1^ rescue plasmid, 3.5 ng·μL^−1^ co‐injection marker plasmid pCFJ90 (Pmyo‐2::mCherry::unc‐54utr, expression causes red fluorescent pharynx), 75 ng·μL^−1^ salmon sperm DNA (Merck, Burlington, MA, USA)] was injected into the gonad of young adult worms using a Zeiss Axiovert S 100 injection microscope. After injection, the worms were washed off the injection pad using M9 buffer and put back on seeded 60 mm NGM plates to recover. The F1 generation was then screened for transformed worms expressing the co‐injection marker under the binocular fluorescence microscope. The F1 worms with a red pharynx were put on separate plates. These plates were then checked for inheritable expression (stable transgenic lines).

### Offspring/embryonic lethality assay

10 L1 worms were moved to 60 mm NGM plates and incubated at the indicated temperature. As soon as egg laying started, they were moved to a new plate every day. One day after the mothers have been moved to a new plate, the hatched worms and dead eggs on the old plate were counted. This was repeated until egg laying stopped.

*af5*/*tm5332* worms were produced by crossing *af5* hermaphrodites with *tm5332* males. After completion of the assay diagnostic PCR (described above) was used to confirm their heterozygosity.

For the rescue experiment with wild‐type sperm, always 5 hermaphrodites (*af5* or *tm5332*) grown at 26 °C were incubated with 10 wild‐type males which were grown at 20 °C. The wild‐type males were added to the hermaphrodite plate as soon as the hermaphrodites reached the L4 stage and then transferred to new plates together with the hermaphrodites during the assay.

### DAPI staining of spermatheca and imaging

Adult worms grown at permissive temperature were left on a 60 mm NGM plate to lay eggs for 2 h. The synchronized eggs on the plate were then incubated at 26 °C for 64 h. The now young adult worms (just started egg laying) were picked into a drop of 3% PFA on a depression slide and incubated for 1 h at room temperature. After washing the worms twice in TBST for 5 min, they were fixed in ice‐cold methanol for 10 min at −20 °C. After again washing the worms twice in TBST for 5 min, the worms were stained in 1 μg·mL^−1^ DAPI in TBST for 10 min. The worms were then mounted in citifluor. The coverslip was sealed using nail polish. The DAPI staining protocol was adapted from Francis *et al*. [29].

Images were captured using an ORCA‐Flash4.0 V3 Digital CMOS camera (Hamamatsu Photonics, Hamamatsu, Japan) mounted on a Axio Imager.M2 (ZEISS) using the Plan‐APOCHROMAT 63x/1.4 Oil objective (ZEISS). The DAPI filter cube “filter set 02” (Item number 488002‐9901‐000, ZEISS) was used. The imaging system was controlled by zen 2.6 pro software (ZEISS).

The number of sperms per in each spermatheca was counted manually using the built‐in multi‐point tool in fiji [30].

### AlphaFold2 modeling

Structure prediction of Y71F9AL.4/PPH‐7 was performed using alphafold2 [31] on the COSMIC^2^ platform [32] (Parameters: Database = full_dbs, Generate predicted template modeling (pTM) score = True, Skip calculating relaxed models = True).

### Sample preparation for proteome/phosphoproteome analysis

100 L4 worms/sample were incubated at 20 °C for 2 days. After 2 days, these day 2 adult worms were placed on 4 new 60 mm NGM plates (25 worms per plate) and incubated at 20 °C for 5–7 h until they laid 500–600 eggs. After removing the worms, the plates full of eggs were incubated at 26 °C for 53–55 h until the worms had reached the young adult stage. The worms were then washed off using M9, washed 3 times in M9 and once in lysis buffer and flash frozen immediately in liquid nitrogen.

### *C. elegans* phosphoproteome analysis

Worms were resuspended in lysis buffer (5% SDS, 10 mm TCEP, 0.1 m TEAB) and lysed by sonication using a PIXUL Multi‐Sample Sonicator (Active Motif, Carlsbad, CA, USA) with Pulse set to 50, PRF to 1, Process Time to 20 min and Burst Rate to 20 Hz followed by a 10 min incubation at 95 °C. Lysates were TCA precipitated according to a protocol originally from Luis Sanchez (https://www.its.caltech.edu/~bjorker/TCA_ppt_protocol.pdf) as follows. One volume of TCA was added to every 4 volumes of sample, mixed by vortexing, incubated for 10 min at 4 °C followed by collection of precipitate by centrifugation for 5 min at 23 000 ***g***. Supernatant was discarded, pellets were washed twice with acetone precooled to −20 °C and the washed pellets were incubated open at RT for 1 min to allow residual acetone to evaporate. Pellets were resuspended in 2 m guanidinium HCl, 0.1 m ammonium bicarbonate, 5 mm Tris (2‐carboxyethyl) phosphine hydrochloride solution (TCEP), phosphatase inhibitors (Sigma P5726&P0044) and proteins were digested as described previously [33]. Shortly, proteins were reduced with 5 mm TCEP for 60 min at 37 °C and alkylated with 10 mm chloroacetamide for 30 min at 37 °C. After diluting samples with 0.1 m ammonium bicarbonate buffer to a final guanidinium HCl concentration of 0.4 m, proteins were digested by incubation with sequencing‐grade modified trypsin (1/100, w/w; Promega, Madison, WI, USA) for 12 h at 37 °C. After acidification using 5% TFA, peptides were desalted using C18 reverse‐phase spin columns (Macrospin, Harvard Apparatus, Holliston, MA, USA) according to the manufacturer's instructions, dried under vacuum and stored at −20 °C until further use.

Peptide samples were enriched for phosphorylated peptides using Fe(III)‐IMAC cartridges on an AssayMAP Bravo platform as recently described [34]. Both the enriched phosphopeptides as well as the unphosphorylated peptides (the flowthrough) were dried under vaccum and stored at −20 °C until further use.

Phospho‐enriched peptides were resuspended in 0.1% aqueous formic acid and subjected to LC–MS/MS analysis using a Orbitrap Fusion Lumos Mass Spectrometer fitted with an EASY‐nLC 1200 (both Thermo Fisher Scientific, Waltham, MA, USA) and a custom‐made column heater set to 60 °C. Peptides were resolved using a RP‐HPLC column (75 μm × 36 cm) packed in‐house with C18 resin (ReproSil‐Pur C18–AQ, 1.9 μm resin; Dr. Maisch GmbH, Ammerbuch, Germany) at a flow rate of 0.2 μL·min^−1^. The following gradient was used for peptide separation: from 5% B to 8% B over 5 min to 20% B over 45 min to 25% B over 15 min to 30% B over 10 min to 35% B over 7 min to 42% B over 5 min to 50% B over 3 min to 95% B over 2 min followed by 18 min at 95% B. Buffer A was 0.1% formic acid in water and buffer B was 80% acetonitrile, 0.1% formic acid in water.

The mass spectrometer was operated in DDA mode with a cycle time of 3 s between master scans. Each master scan was acquired in the Orbitrap at a resolution of 120 000 FWHM (at 200 *m*/*z*) and a scan range from 375 to 1600 *m*/*z* followed by MS2 scans of the most intense precursors in the Orbitrap at a resolution of 30 000 FWHM (at 200 *m*/*z*) with isolation width of the quadrupole set to 1.4 *m*/*z*. Maximum ion injection time was set to 50 ms (MS1) and 54 ms (MS2) with normalized AGC target set to 250% and 100%, respectively. Only peptides with charge state 2–5 were included in the analysis. Monoisotopic precursor selection (MIPS) was set to Peptide, and the Intensity Threshold was set to 2.5e4. Peptides were fragmented by HCD (higher energy collisional dissociation) with collision energy set to 30%, and one microscan was acquired for each spectrum. The dynamic exclusion duration was set to 30 s.

The acquired raw files were imported into the progenesis qi software (v2.0, Nonlinear Dynamics Limited, Milford, MA, USA), which was used to extract peptide precursor ion intensities across all samples applying the default parameters. The generated mgf‐file was searched using MASCOT against a *C. elegans* database (consisting of 56 956 forward and reverse protein sequences downloaded from Uniprot on 20200421) and 392 commonly observed contaminants using the following search criteria: full tryptic specificity was required (cleavage after lysine or arginine residues, unless followed by proline); 3 missed cleavages were allowed; carbamidomethylation (C) was set as fixed modification; oxidation (M) and phosphorylation (STY) were applied as variable modifications; mass tolerance of 10 ppm (precursor) and 0.02 Da (fragments). The database search results were filtered using the ion score to set the false discovery rate (FDR) to 1% on the peptide and protein level, respectively, based on the number of reverse protein sequence hits in the datasets. Quantitative analysis results from label‐free quantification were processed using the safequant r package v.2.3.2. ([33], https://github.com/eahrne/SafeQuant/) to obtain peptide relative abundances. This analysis included global data normalization by equalizing the total peak/reporter areas across all LC–MS runs, data imputation using the knn algorithm, summation of peak areas per LC–MS/MS run, followed by calculation of peptide abundance ratios. Only isoform specific peptide ion signals were considered for Quantification. To meet additional assumptions (normality and homoscedasticity) underlying the use of linear regression models and t‐tests, MS‐intensity signals were transformed from the linear to the log‐scale. The summarized peptide expression values were used for statistical testing of between condition differentially abundant peptides. Here, empirical Bayes moderated t‐tests were applied, as implemented in the R/Bioconductor limma package (http://bioconductor.org/packages/release/bioc/html/limma.html) were used.

### *C. elegans* proteome analysis

Dried peptides (flowthrough from phospho‐peptide enrichment described above) were resuspended in 0.1% aqueous formic acid and subjected to LC–MS/MS analysis using a Exploris 480 Mass Spectrometer fitted with an Vanquish Neo (both Thermo Fisher Scientific) and a custom‐made column heater set to 60 °C. Peptides were resolved using a RP‐HPLC column (75 μm × 30 cm) packed in‐house with C18 resin (ReproSil‐Pur C18–AQ, 1.9 μm resin; Dr. Maisch GmbH) at a flow rate of 0.2 μL·min^−1^. The following gradient was used for peptide separation: from 4% B to 10% B over 5 min to 35% B over 45 min to 50% B over 10 min to 95% B over 1 min followed by 10 min at 95% B to 5% B over 1 min followed by 4 min at 5% B. Buffer A was 0.1% formic acid in water and buffer B was 80% acetonitrile, 0.1% formic acid in water.

The mass spectrometer was operated in DIA mode with a cycle time of 3 s. MS1 scans were acquired in the Orbitrap in centroid mode at a resolution of 120 000 FWHM (at 200 *m*/*z*), a scan range from 390 to 910 *m*/*z*, normalized AGC target set to 300% and maximum ion injection time mode set to Auto. MS2 scans were acquired in the Orbitrap in centroid mode at a resolution of 15 000 FWHM (at 200 *m*/*z*), precursor mass range of 400–900, quadrupole isolation window of 12 *m*/*z* with 1 *m*/*z* window overlap, a defined first mass of 120 *m*/*z*, normalized AGC target set to 3000% and a maximum injection time of 22 ms. Peptides were fragmented by HCD (Higher‐energy collisional dissociation) with collision energy set to 28% and one microscan was acquired for each spectrum.

The acquired raw files were searched using the spectronaut (Biognosys v17.3, Schlieren, Switzerland) directDIA workflow against a *C. elegans* database (consisting of 26 585 protein sequences downloaded from Uniprot on 20220222) and 392 commonly observed contaminants. Quantitative data was exported from Spectronaut and analyzed using the msstats r package v.4.7.2. (https://doi.org/10.1093/bioinformatics/btu305).

## Results

### FA50 is a temperature‐sensitive mutant, which carries a 2‐bp deletion on chromosome I

**Fig. 1:**
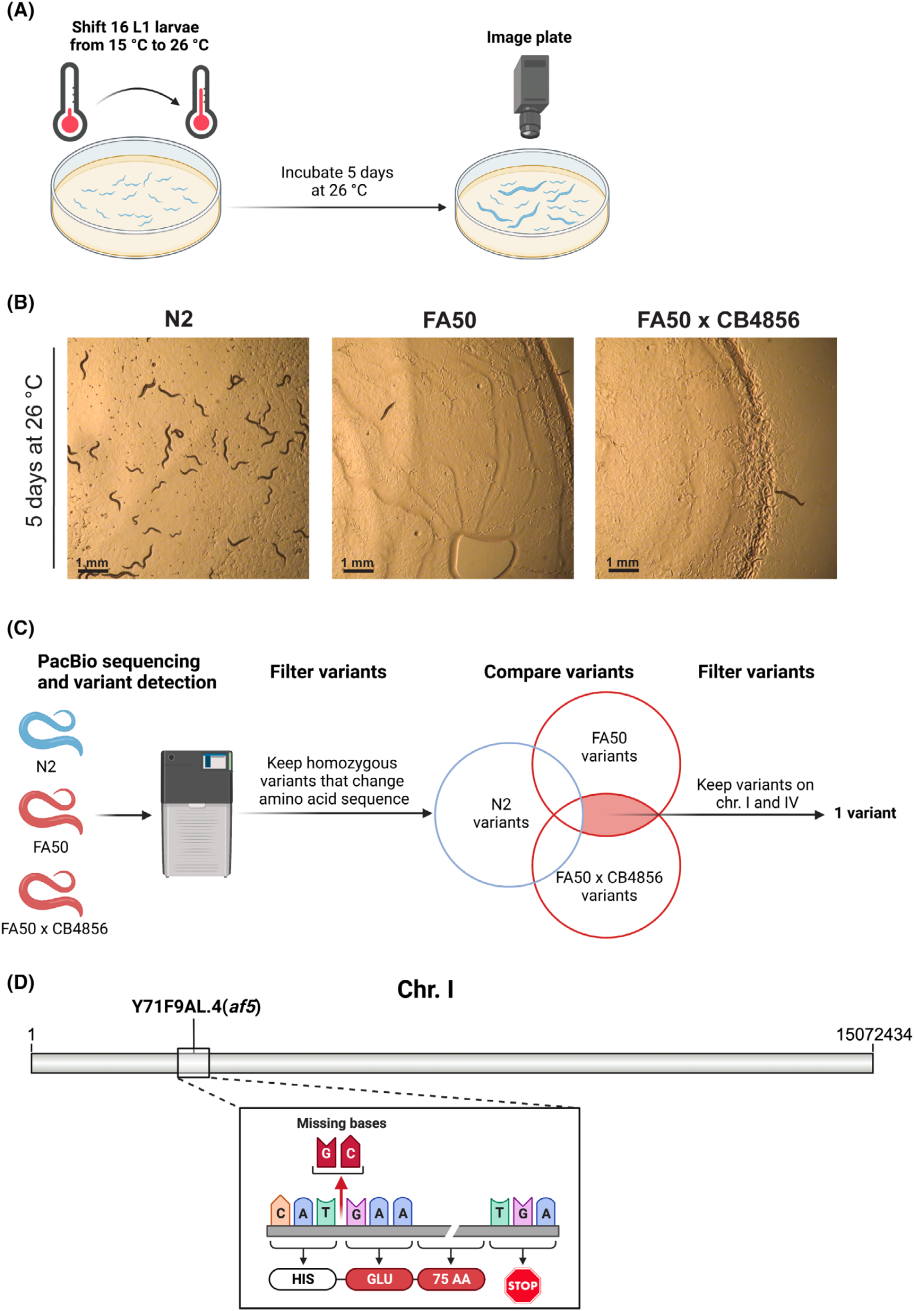
FA50 is a temperature‐sensitive mutant which carries a 2 bp deletion on chromosome I. (A) Standard protocol used to image temperature‐sensitive phenotypes. Temperature‐sensitive strains show a reduced number of worms and eggs on the plate after 5 days. (B) N2 wild type, FA50 and FA50 × CB4856 strain after 5 days incubation at 26 °C. Scale bar: 1 mm. (C) Sequencing and data analysis pipeline used to identify mutations shared between the two FA50 strains. (D) 2‐bp deletion (*af5*) identified in the Y71F9AL.4 gene on chromosome I. The deletion causes a frameshift resulting in a premature stop codon 76 amino acids after the frameshift.

Over two decades ago, in an ethyl methane sulfonate (EMS) screen for temperature‐sensitive embryonic lethality in *C. elegans* we isolated a mutant, FA50, carrying the *af5* allele. Some initial characterization and mapping attempts were performed at the time, but because of the rather time‐consuming and cumbersome cloning of mutants, *af5* was never cloned. Given the advances in technology, we recently set out to identify the lesion in FA50. To do this, we first verified the phenotype by shifting L1 larvae from the permissive temperature (15 °C) to the restrictive temperature (26 °C) for five days (Fig. 1A). While N2 wild‐type animals grew and generated offspring at the expected rate, FA50 mutant animals reached adulthood, but very few eggs were observed, and the eggs present on the plate failed to hatch (Fig. 1B).

When we initially isolated the FA50 mutant, we aimed to map the lesion by single nucleotide polymorphism (SNP) mapping. Therefore, we crossed FA50 to the Hawaiian isolate CB4856. The F3 progeny was used for bulk SNP analysis and revealed that the mutation most likely mapped to chromosome I or IV (Fig. S1A, Table S1). However, the exact location of the lesion remained elusive. From further mapping attempts, clonal FA50 x CB4856 animals were kept as frozen stocks. After shifting FA50 x CB4856 animals to the restrictive temperature, they mirrored the phenotype of FA50 (Fig. 1B).

To identify the mutation responsible for the reduced brood size and embryonic lethality, we decided to isolate genomic DNA from the wild type and the two FA50 mutant strains for PacBio long‐read sequencing (Fig. 1C). We focused on homozygous mutations that would result in amino acid changes in coding sequences. These mutations should be conserved between FA50 and FA50 x CB4856. Within these constraints, we identified only one single 2‐bp deletion in the gene Y71F9AL.4 on chromosome I, which results in a frameshift and a premature stop codon 76 amino acids downstream of the lesion (Fig. 1D). No relevant mutations were observed on chromosome IV. These data indicate that a 2‐bp deletion, which causes a frameshift in the Y71F9AL.4 gene on chromosome I might be responsible for the temperature‐sensitive fertility defect and embryonic lethality observed in FA50 mutant animals.

### The phenotype in FA50 is caused by a 2‐bp deletion in Y71F9AL.4

**Fig. 2:**
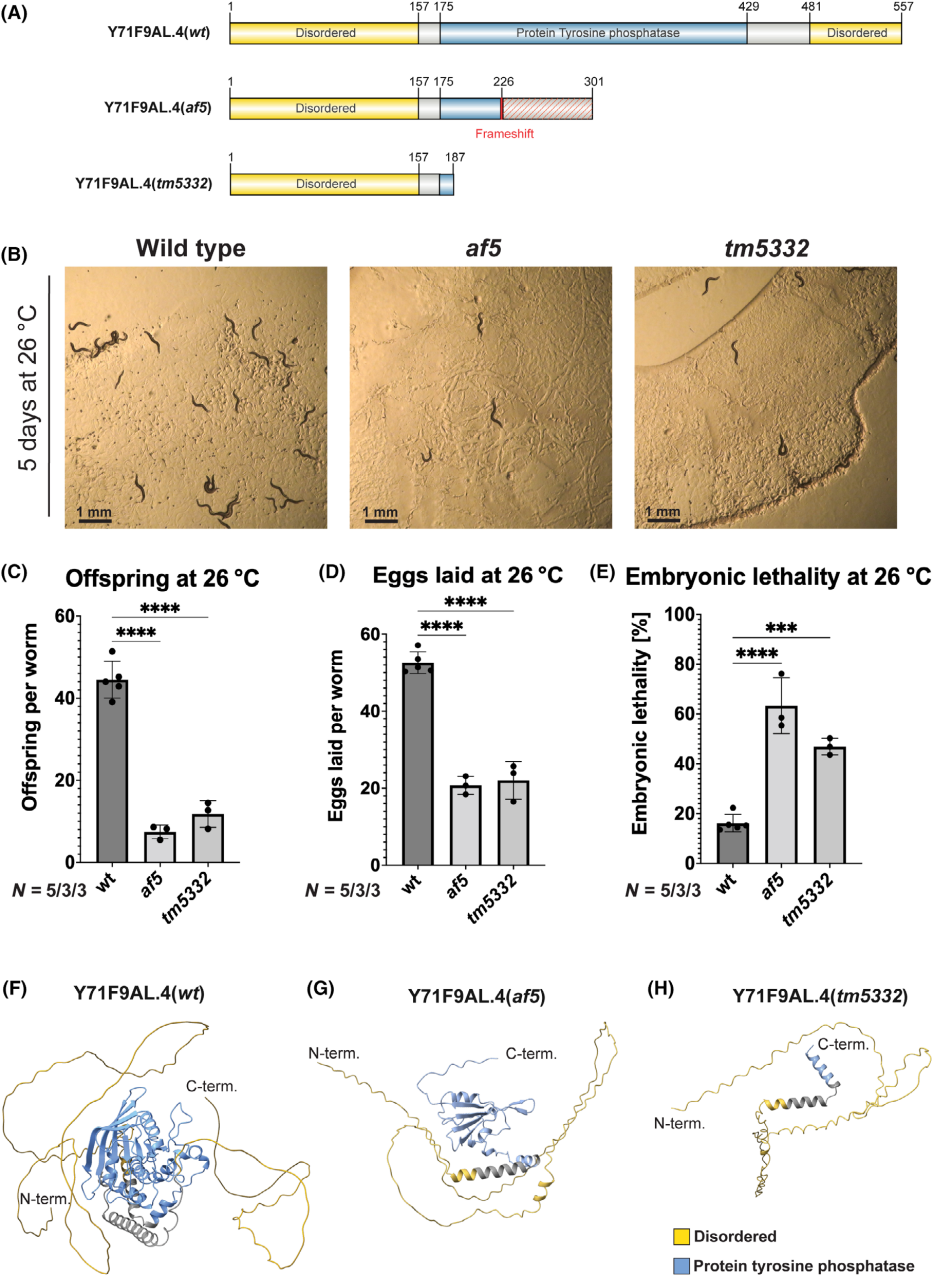
Y71F9AL.4 is a protein tyrosine phosphatase required for fertility and embryonic development at elevated temperatures. (A) Schematic of amino acid sequence for native Y71F9AL.4 and the truncated versions Y71F9AL.4(*af5*) and Y71F9AL.4(*tm5332*). (B) Wild type, *af5* and *tm5332* worms after 5 days incubation at 26 °C. Scale bar: 1 mm. (C) Offspring at 26 °C. Number of offspring produced per worm at 26 °C was determined for *n* = 10 worms per experiment for *N* = 5 (wild type), *N* = 3 (af5 and tm5332) biological replicates. Data are presented as mean ± SD. Statistical significance was determined using one‐way ANOVA: ****P ≤ 0.0001. (D) Eggs laid at 26 °C. Number of eggs laid per worm at 26 °C was determined for *n* = 10 worms per experiment for *N* = 5 (wild type), *N* = 3 (af5 and tm5332) biological replicates. Data are presented as mean ± SD. Statistical significance was determined using one‐way ANOVA: ****P ≤ 0.0001. (E) Embryonic lethality at 26 °C. Percentage of eggs that did not hatch was determined for *n* = 10 worms per experiment for *N* = 5 (wild type), *N* = 3 (af5 and tm5332) biological replicates. Data are presented as mean ± SD. Statistical significance was determined using one‐way ANOVA: ***P ≤ 0.001, ****P ≤ 0.0001. (F) AlphaFold2 predictions of native Y71F9AL.4. The PTP‐domain is flanked by two disordered domains. (G, H) AlphaFold2 predictions of the truncated versions Y71F9AL.4(*af5*) (G) and Y71F9AL.4(*tm5332*) (H).

**Fig. 3:**
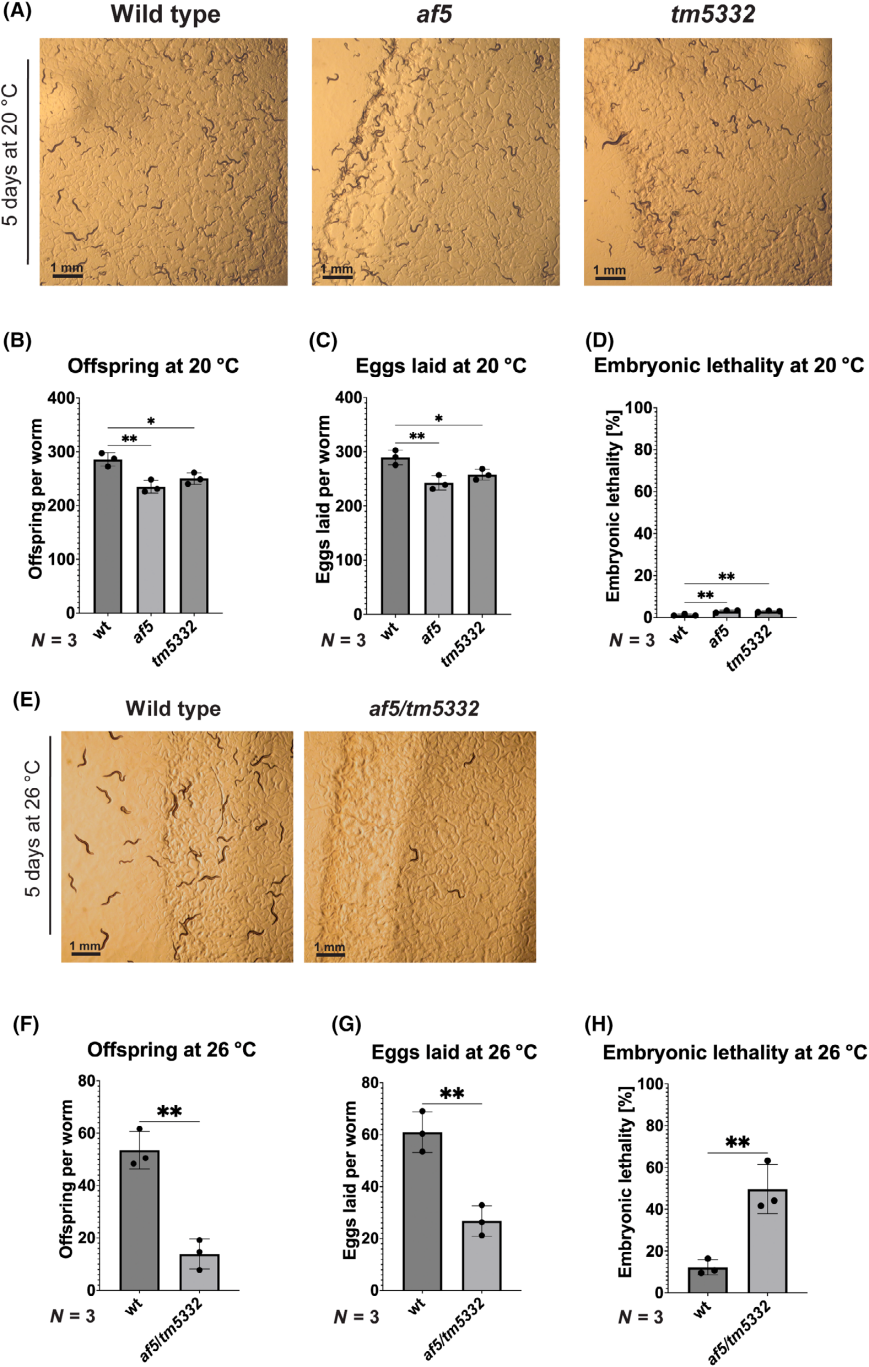
Fertility defects are minor at 20 °C and specific to the Y71F9AL.4 locus. (A) Wild type, *af5* and *tm5332* worms after 5 days incubation at 20 °C. Scale bar: 1 mm. (B) Offspring at 20 °C. Number of offspring produced per worm at 20 °C was determined for *n* = 10 worms per experiment for *N* = 3 biological replicates. Data are presented as mean ± SD. Statistical significance was determined using one‐way ANOVA: *P ≤ 0.05, **P ≤ 0.01. (C) Eggs laid at 20 °C. Number of eggs laid per worm at 20 °C was determined for *n* = 10 worms per experiment for *N* = 3 biological replicates. Data are presented as mean ± SD. Statistical significance was determined using one‐way ANOVA: *P ≤ 0.05, **P ≤ 0.01. (D) Embryonic lethality at 20 °C. Percentage of eggs that did not hatch was determined for *n* = 10 worms per experiment for *N* = 3 biological replicates. Data are presented as mean ± SD. Statistical significance was determined using one‐way ANOVA: **P ≤ 0.01. (E) Wild type and *af5*/*tm5332* worms after 5 days incubation at 26 °C. Scale bar: 1 mm. (F) Offspring at 26 °C. Number of offspring produced per worm at 26 °C was determined for *n* = 10 worms per experiment for *N* = 3 biological replicates. Data are presented as mean ± SD. Statistical significance was determined using unpaired two‐tailed *t* test: **P ≤ 0.01. (G) Eggs laid at 26 °C. Number of eggs laid per worm at 26 °C was determined for *n* = 10 worms per experiment for *N* = 3 biological replicates. Data are presented as mean ± SD. Statistical significance was determined using unpaired two‐tailed *t* test: **P ≤ 0.01. (H) Embryonic lethality at 26 °C. Percentage of eggs that did not hatch was determined for *n* = 10 worms per experiment for *N* = 3 biological replicates. Data are presented as mean ± SD. Statistical significance was determined using unpaired two‐tailed *t* test: **P ≤ 0.01.

A knockout allele of Y71F9AL.4, *tm5332*, was created by the National Bioresource Project in Japan as a part of the International *C. elegans* Gene Knockout consortium but had not been characterized (Fig. 2A) [35]. To ensure that the 2‐bp lesion found in FA50 (*af5*) (Fig. 2A) is indeed responsible for the temperature‐sensitive embryonic lethal phenotype, we determined the phenotype of the *tm5332* and compared it to the *af5* allele. First, we backcrossed the two mutant alleles *af5* and *tm5332* twice and four times, respectively, to remove potential secondary mutations. Similar to the *af5* allele, we observed a strong reduction in offspring in *tm5332* upon shift to the restrictive temperature (Fig. 2B,C). This reduction in offspring was due to the limited number of eggs laid and to the failure of the embryos to develop into larvae in both mutants (Fig. 2D,E). The differences in the number of eggs laid were especially pronounced within the first 24 h of egg laying (Fig. S1B). Although *af5* and *tm5332* mutants grown at 20 °C did not show any obvious phenotypes, quantification revealed that mutants grown at permissive temperature also exhibit minor fertility defects (Fig. 3A–D). We did not observe any obvious accumulation of unfertilized oocytes on the plates. At the restrictive temperature, the embryos did not arrest at any particular stage during embryonic development, indicating that Y71F9AL.4 is not required at a specific stage during embryogenesis. Our data are consistent with the notion that the fertility and embryonic lethal phenotypes in FA50 (*af5*) are caused by the 2‐bp deletion in Y71F9AL.4. We aimed to rescue the phenotype of *af5* or *tm5332* by re‐expression of wild‐type Y71F9AL.4. We were unable, however, to propagate transgenic lines generated by microinjection with or without fluorescent tags in mutant or wild‐type animals (Fig. S1C). From these negative data, we conclude that overexpression of Y71F9AL.4 might be detrimental for survival of the worms. Therefore, we decided to determine the fertility of *af5*/*tm5332* hermaphrodites at 26 °C. *af5*/*tm5332* hermaphrodites phenocopy the worms carrying the individual alleles (Fig. 3E–H), confirming that the observed phenotype is indeed specific to the Y71F9AL.4 locus. Taken together, our data provide strong evidence that loss of function of Y71F9AL.4 in *af5* and *tm5332* results in reduced fertility and embryonic lethality at 26 °C.

Y71F9AL.4 is mostly expressed in the ectoderm during embryonic development and is enriched in neurons and germline at larval and adult stages [36, 37, 38]. This expression pattern appears to be consistent with the observed phenotype of reduced fertility and embryonic lethality. In addition, Y71F9AL.4 seems to be more highly expressed in males than in hermaphrodites [39].

### Y71F9AL.4 encodes a conserved protein tyrosine phosphatase

Y71F9AL.4 encodes an uncharacterized 557‐amino acid protein carrying a protein tyrosine phosphatase domain flanked by two disordered domains (Fig. 2A,F) [40]. We used Alpha Fold to predict the structure of the protein of the *af5* and *tm5532* alleles [31]. For Y71F9AL.4 (*af5*) a part of the phosphatase domain was lost, while it was completely absent in Y71F9AL.4 (*tm5332*) (Fig. 2G,H). We next aimed to determine whether any truncated versions of Y71F9AL.4 was expressed in the worm. To this end, we expressed the N‐terminal part of Y71F9AL.4 in *E. coli* and used the enriched proteins for antibody production. Unfortunately, no specific antibodies could be obtained. Thus, we cannot exclude that a truncated version of Y71F9AL.4 is expressed in *af5*, but based on our phenotypic analysis, we would predict that this truncated protein would not be functional.

**Fig. 4:**
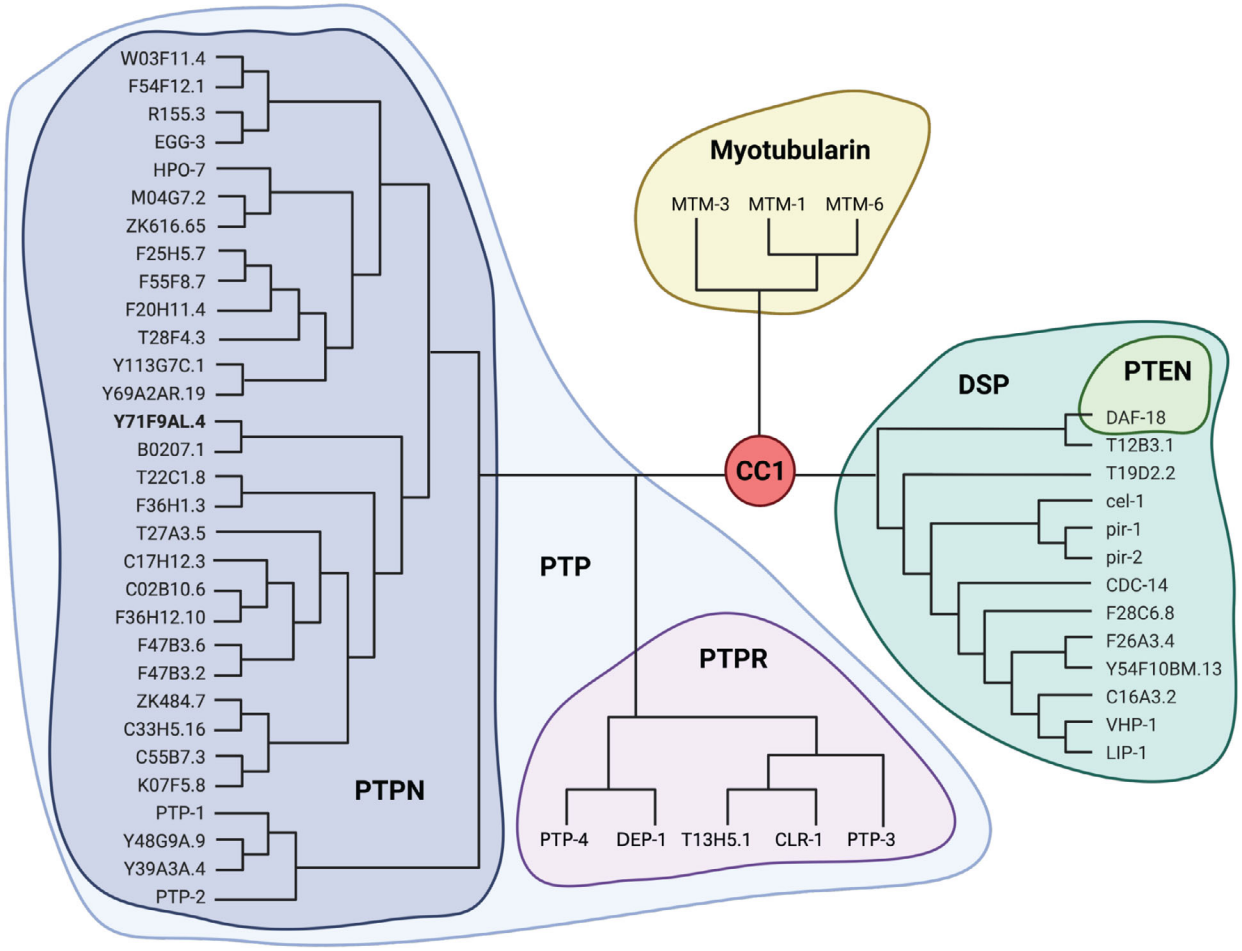
Y71F9AL.4 belongs to the PTPN gene family. Phylogenetic tree of all active cysteine‐based class I (CC1) phosphatases in *C. elegans*. Tree is based on a multiple sequence alignment of all catalytically active CC1 phosphatases [11] performed using the clustal omega program [62]. Families/subfamilies shown in color: Protein tyrosine phosphatases (PTP), receptor type PTPs (PTPR), dual specificity phosphatases (DSP), phosphatase and tensin homologs (PTEN) and myotubularins. Y71F9AL.4 belongs to the PTPN gene family and is shown in bold.

**Fig. 5:**
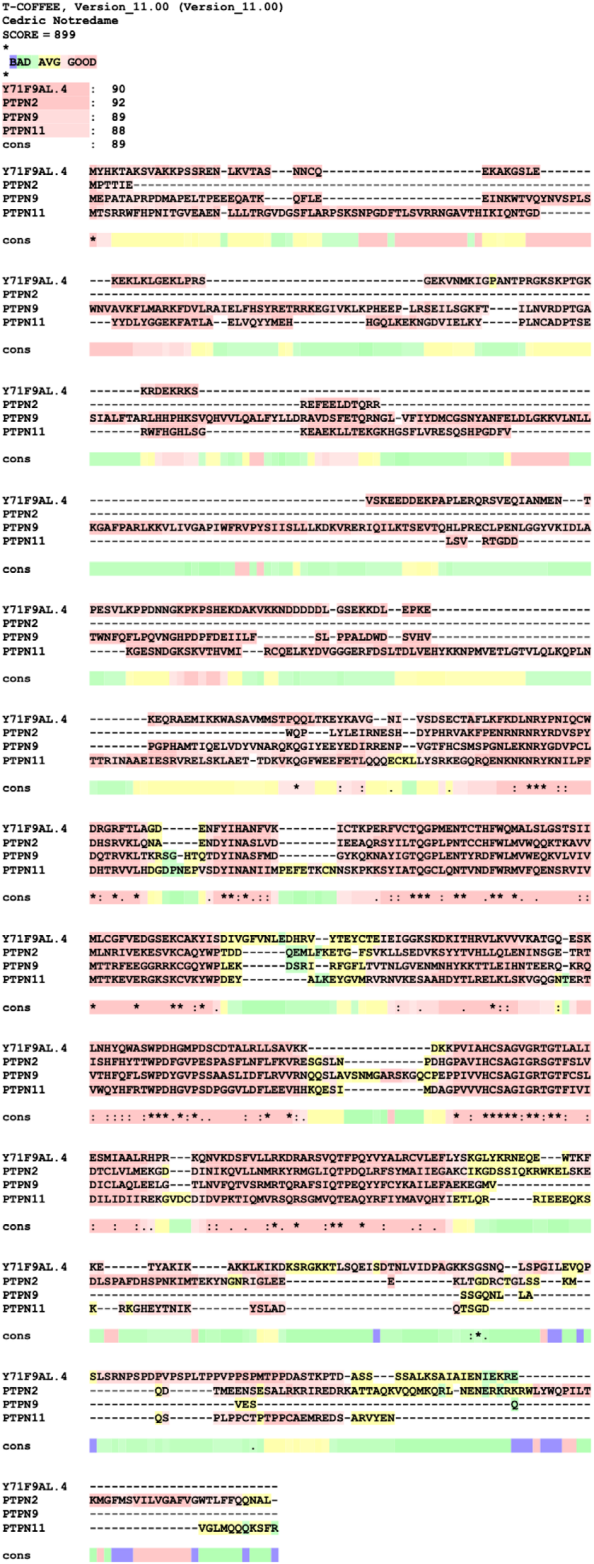
The closest human homologs of Y71F9AL.4 are PTPN2/9/11. Multiple sequence alignment of Y71F9AL.4 and the human PTPN2/9/11 using T‐COFFEE [63, 64]. Red stretches indicate good conservation.

The protein tyrosine phosphatase domain of Y71F9AL.4 is homologous to the phosphatase domain of the human PTPN protein family (Fig. 4). This family contains 31 members in *C. elegans* and 17 members in humans [41, 42]. Unfortunately, the conservation outside the phosphatase domain is not high enough to identify the direct corresponding homolog. Depending on the software used PTPN1, PTPN2, PTPN9 or PTPN11 were identified as closest human homologs (Fig. 5) [41, 43]. Since we could not identify a single mammalian homolog of Y71F9AL.4, we named it in accordance with the *C. elegans* nomenclature protein phosphatase 7, PPH‐7.

### *pph‐7* mutants exhibit spermatogenesis defects

**Fig. 6:**
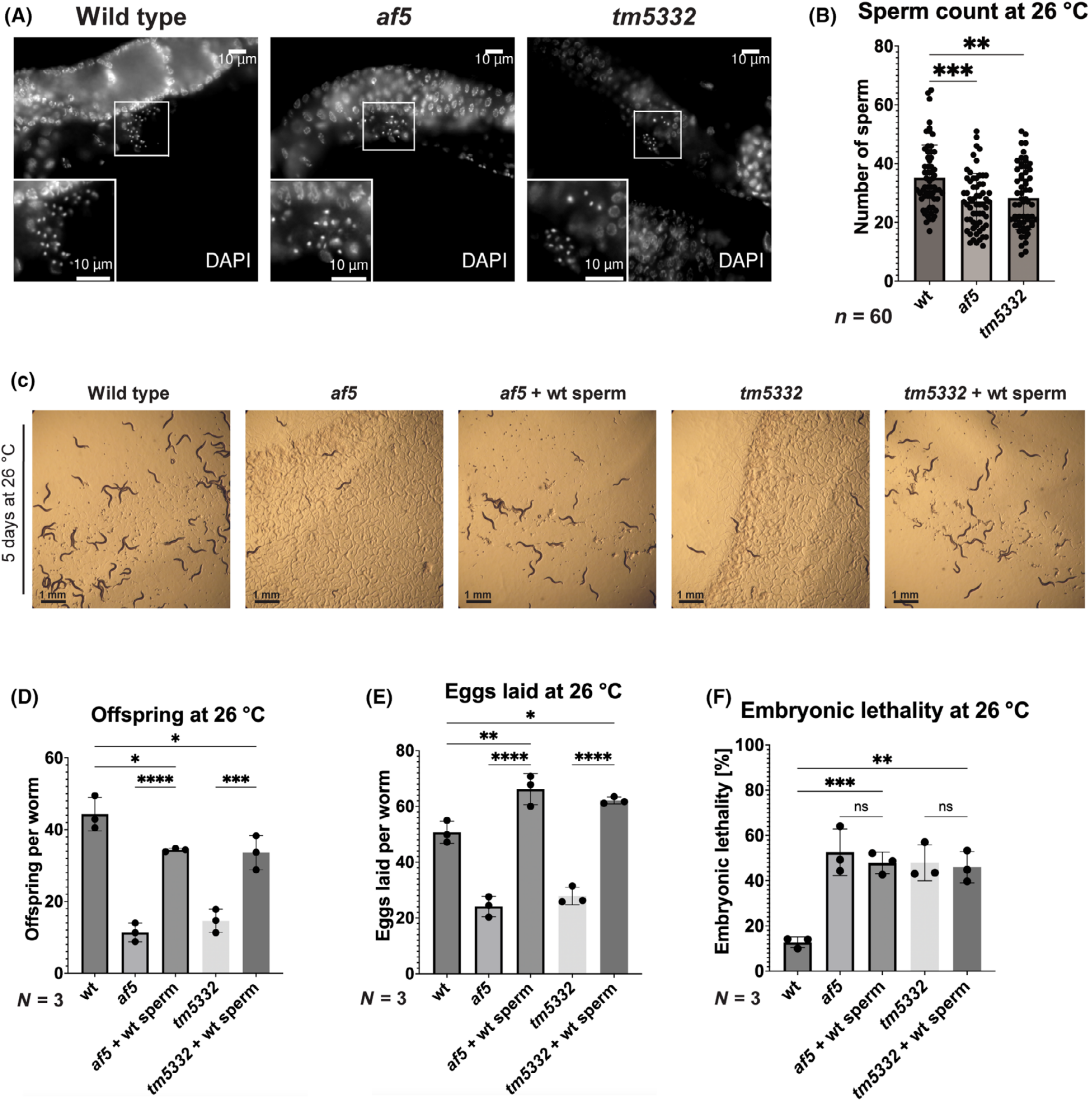
*pph‐7* mutants exhibit defects in spermatogenesis. (A) DAPI‐stained wild type, *af5* and *tm5332* worms. The enlargement shows sperm contained in the spermatheca. Worms were grown at 26 °C, fixed, DAPI stained and imaged using a brightfield microscope. Scale bar: 10 μm. (B) Quantification of the sperm number in wild type, *af5* and *tm5332* worms based on images taken in A. Images of *n* = 20 worms were quantified for *N* = 3 biological replicates. Data are presented as mean ± SD. Statistical significance was determined using one‐way ANOVA: **P ≤ 0.01, ***P ≤ 0.001. (C) Wild type, *af5*, *tm5332* worms after 5 days incubation at 26 °C. In the *af5* + wt sperm and *tm5332* + wt sperm panels, wild‐type males were added to the plate to provide the hermaphrodites with functional sperm. Scale bar: 1 mm. (D) Offspring at 26 °C. Number of offspring produced per worm at 26 °C was determined for *n* = 10 worms per experiment for *N* = 3 biological replicates. Data are presented as mean ± SD. Statistical significance was determined using one‐way ANOVA: *P ≤ 0.05, ***P ≤ 0.001, ****P ≤ 0.0001. (E) Eggs laid at 26 °C. Number of eggs laid per worm at 26 °C was determined for *n* = 10 worms per experiment for *N* = 3 biological replicates. Data are presented as mean ± SD. Statistical significance was determined using one‐way ANOVA: *P ≤ 0.05, **P ≤ 0.01, ****P ≤ 0.0001. (F) Embryonic lethality at 26 °C. Percentage of eggs that did not hatch was determined for *n* = 10 worms per experiment for *N* = 3 biological replicates. Data are presented as mean ± SD. Statistical significance was determined using one‐way ANOVA: **P ≤ 0.01, ***P ≤ 0.001.

Due to the high expression of *pph‐7* in males and in the germline, we decided to investigate sperm number and function in *pph‐7* mutants as potential sources of the observed fertility defects. In order determine the sperm number, we performed a whole body DAPI staining of fixed wild type and *pph‐7* mutant hermaphrodites grown at 26 °C and counted the number of sperm present in the spermatheca (Fig. 6A,B). We observed a small but significant reduction in the number of sperm in *pph‐7* mutant worms. This reduction in the number of sperm could result from fewer sperm produced or the inability of the sperm to maintain position in the spermatheca [44]. No obvious increase in the number of sperm spread throughout the uterus was observed suggesting that the number of sperm produced is smaller in *pph‐7* mutants. However, we deemed it unlikely that this small reduction is the sole cause of the observed fertility defects. Thus, we next investigated sperm function by providing *pph‐7* mutant hermaphrodites grown at 26 °C with functional sperm from wild‐type males and determining the number of offspring produced. We observed a strong increase in the number of viable offspring when *pph‐7* mutants were provided with wild‐type sperm (Fig. 6C,D). This increase was due to a strong increase in the number of eggs laid (Fig. 6E), suggesting that the observed reduction of brood size in *pph‐7* worms is indeed due to spermatogenesis‐related defects. However, we did not see a reduction in the embryonic lethality when wild‐type sperm was provided to *pph‐7* mutant hermaphrodites (Fig. 6F), indicating that *pph‐7* is also required maternally for embryonic development. Taken together, our findings indicate a role of functional PPH‐7 for spermatogenesis at elevated temperatures, with a possible additional function in development.

### PPH‐7 regulates targets of the von Hippel–Lindau tumor suppressor pathway

**Fig. 7:**
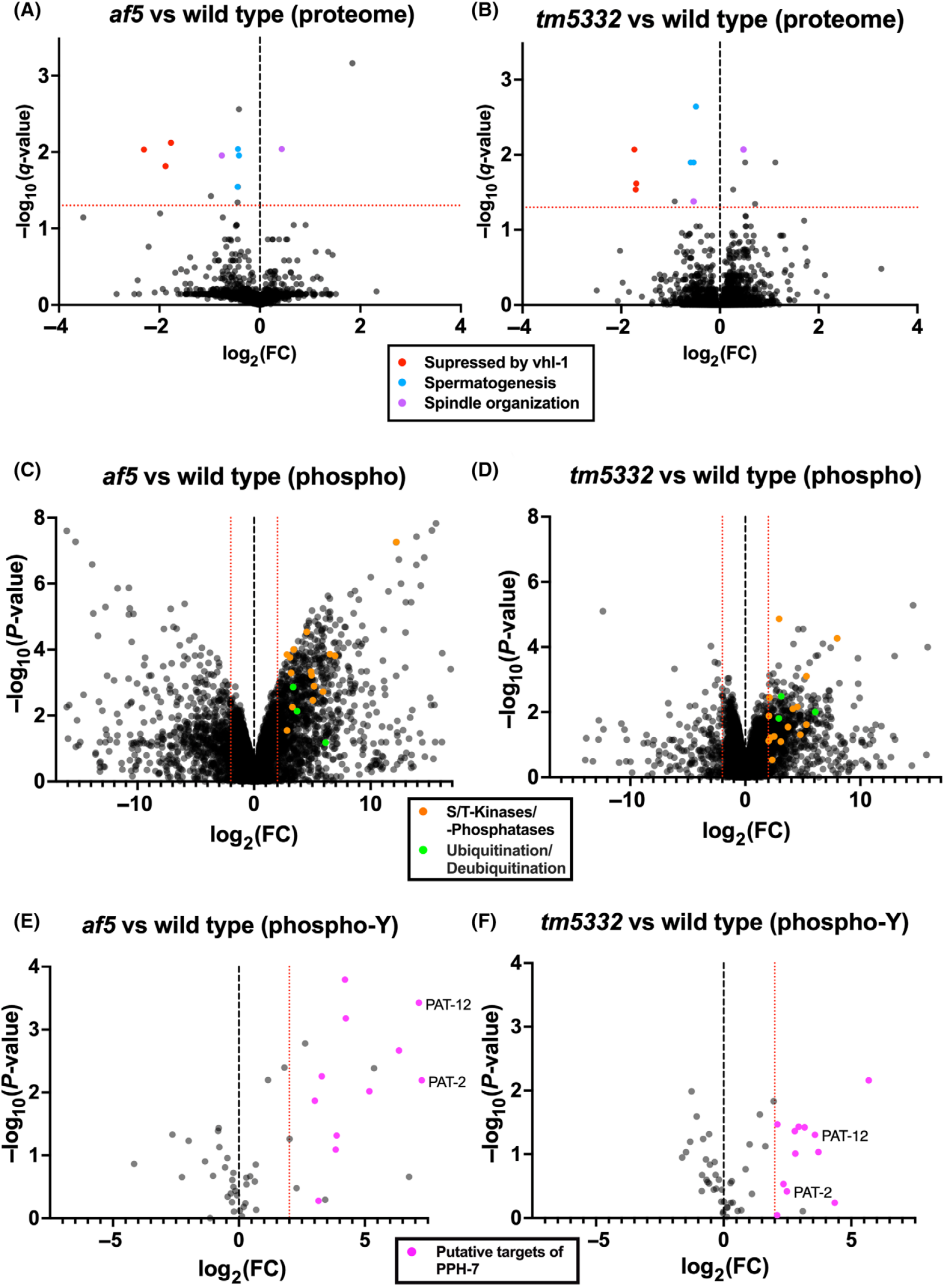
PPH‐7 modulates the levels spermatogenesis proteins and *vhl‐1* targets. (A, B) Volcano plots showing all proteins identified using a LC–MS/MS approach for *af5* vs wild type (A) and *tm5332* vs wild type (B). *X*‐axis indicates the log_2_(fold change) and the *Y*‐axis indicates the −log_10_(*q*‐value). Proteins above the red dotted line (*q*‐value ≤ 0.05) show significantly different levels in the mutants compared to wild type. Related groups of proteins are highlighted in red (suppressed by *vhl‐1*), blue (spermatogenesis) and purple (spindle organization). (C, D) Volcano plots showing all phospho‐peptides identified for *af5* vs wild type (C) and *tm5332* vs wild type (D). *X*‐axis indicates the log_2_(fold change) and the *Y*‐axis indicates the −log_10_(*P*‐value). Phospho‐peptides belonging to related groups of proteins are highlighted in orange (serine/threonine kinases and phosphatases) and green (ubiquitination/deubiquitination). Red dotted lines indicate a log_2_(FC) = ± 2. (E, F) Volcano plots showing all proteins phosphorylated on a tyrosine residue identified for *af5* vs wild type (E) and *tm533*2 vs wild type (F). *X*‐axis indicates the log_2_(fold change) and the *Y*‐axis indicates the −log_10_(*P*‐value). Phospho‐peptides that show a log_2_(fold change) > 2 in *af5* as well as *tm5332* are highlighted in pink (putative targets of PPH‐7). Red dotted line indicates a log_2_(FC) = 2.

From sequence homology searches it was impossible to assign a specific human homolog for PPH‐7, which would also be informative about potential targets of the phosphatase and the molecular pathways involved. To get an understanding about the cellular role of PPH‐7, we performed proteomics and phosphoproteomic analyses in wild‐type, *af5* and *tm5332* animals grown at the restrictive temperature 26 °C. Overall, we detected about 5500 proteins per sample, indicating that we were able to detect about 25% of the *C. elegans* proteome, which is comparable with previous reports [45]. Only a few proteins were significantly different in their expression levels between wild type and the mutants (10 in *af5* and 13 in *tm5332*; Table S2). Intriguingly, nine of these proteins were regulated similarly in both mutants, confirming independently that the *af5* mutant is indeed defective in *pph‐7*. Three of these proteins (SMZ‐1, SSQ‐1, and MSP‐3) belong to a group of proteins predicted to be involved in spermatogenesis [46, 47, 48] (Fig. 7A,B, blue dots), which is consistent with the reported expression in the germline and the observed reduction in fertility. Two differentially regulated proteins (TPXL‐1 and GIP‐2) have been previously shown to be involved in spindle organization [49, 50] (Fig. 7A,B, purple dots). Strikingly, the three most downregulated proteins (CLEC‐209, MCT‐2 and C01B4.6) in *pph‐7* mutants have been previously described to be downregulated by VHL‐1 in a HIF‐1 independent manner (Fig. 7A,B, red dots) [21]. VHL‐1 is the homolog of the human E3 ubiquitin ligase von Hippel–Landau tumor suppressor protein.

In the phosphoproteome, we detected around 6500 phospho‐peptides per sample. We observed broad changes in the phosphorylation state of proteins across the proteome, both on tyrosine as well as serine/threonine residues. We were initially puzzled by these results. However, upon closer inspection, we realized that several serine/threonine kinases and phosphatases changed their phosphorylation status dependent on the presence or absence of PPH‐7 (Fig. 7C,D, orange dots, Table S3). Thus, PPH‐7 appears to broadly affect phosphorylation levels. As a result, the phosphoproteome data were rather noisy, and it became difficult to discriminate between direct or indirect effects on the phosphoproteome. Still, we found components of the ubiquitin degradation machinery differentially phosphorylated, which may suggest a role pf PPH‐7 in the regulation of protein stability (Fig. 7C,D, green dots, Table S3). Focusing our analysis on the increase in tyrosine phosphorylation, we identified multiple proteins that were more phosphorylated in *af5* and *tm5332* compared to wild‐type animals. Proteins, which were at least four‐fold more phosphorylated on the same tyrosine residue in both *pph‐7* mutants were considered putative PPH‐7 targets (Fig. 7E,F, pink dots, Table S4). We identified the putative targets PAT‐12 (component of hemidesmosomes) and PAT‐2 (integrin subunit), which both have been previously linked to ECM function [51, 52].

Taken together, our data suggest that the previously uncharacterized protein tyrosine phosphatase PPH‐7 is required for fertility and embryonic development at elevated temperature and that PPH‐7 could potentially modulate the von Hippel–Lindau tumor suppressor pathway in *C. elegans*.

## Discussion

Here, we report the cloning of a loss of function mutation (*af5*) in Y71F9AL.4 in *C. elegans*, which encodes for a protein tyrosine phosphatase and which we named PPH‐7. Loss of PPH‐7 results in reduced fertility and increased embryonic lethality at the restrictive temperature 26 °C. At permissive temperatures, PPH‐7 deficiency did not result in any obvious phenotype, suggesting that its function only becomes essential at elevated temperatures. It is conceivable that there is some redundancy among the PTPN family members in *C. elegans*, which would largely compensate for the loss of PPH‐7 at normal growth conditions but not at elevated temperatures. Elevated temperatures represent stress for the animal, which is also reflected in the drop in fertility observed in wild‐type animals, which produce about 200–300 offspring at 20 °C but only a fraction of this at 26 °C. This can be explained by spermatogenesis and oogenesis being inherently temperature‐sensitive processes [53, 54]. This inherent temperature sensitivity is also making these processes more vulnerable to genetic perturbations at elevated temperatures [55].

To characterize the nature of the fertility defect in *pph‐7* mutants, we determined sperm number and function in PPH‐7 deficient worms. We observed a decrease in the number of sperm present in the spermatheca and were able to increase brood size by addition of males onto the plate indicating that *pph‐7* mutants indeed exhibit defects in spermatogenesis. We cannot exclude that the presence of males could have additional effects other than providing functional sperm (e.g. imposing additional stress) on the hermaphrodites, which could also modulate fertility.

We performed proteomics analysis to identify proteins that might be differentially expressed in wild‐type and in mutant animals. Intriguingly only a few proteins were detected in this analysis. We used young adults for data collection, in which PPH‐7 should be expressed in the germline. Whole animals were processed for the mass spectrometry analysis, which might have reduced the number of specific proteins differentially expressed, we could potentially detect since PPH‐7 is mainly present in the germline. Nevertheless, we were able to sample about 25% of the *C. elegans* proteome, which is comparable to the previous studies [45, 56]. Importantly, the few differentially expressed proteins, were enriched in targets of the von Hippel–Lindau tumor suppressor, VHL‐1. While we did not detect the major target of VHL‐1, the hypoxia‐inducible transcription factor HIF‐1 [14, 15, 16, 17], we found CLEC‐209, MCT‐2 and C01B4.6, whose protein levels appear to be independent of HIF‐1 [21]. These data indicate that PPH‐7 could be involved in the regulation of VHL‐1 activity.

Furthermore, we observed a significant decrease in protein levels for a small number of additional proteins, which could be potentially linked to the observed phenotypes in *pph‐7* mutants. Three of these proteins (SMZ‐1, SSQ‐1, and MSP‐3) are predicted to be involved in spermatogenesis. SMZ‐1 is required for male spermatocytes to progress through meiosis [46], while SSQ‐1 belongs to the nematode and sperm specific protein family, class Q, which is highly expressed in sperm but whose function remains largely elusive [47]. MSP‐3 is part of the major sperm protein (MSP) family, which is involved in MSP filament formation, enabling sperm motility [57]. It is conceivable that the reduced level of those proteins in *pph‐7* mutants could be linked to the observed reduction in sperm number and function.

Finally, we also observed dysregulation of two proteins which are involved in spindle organization (TPXL‐1 and GIP‐2). TPXL‐1 has been shown to activate and target the Aurora kinase (AIR‐1) to microtubules, which is essential for mitotic spindle assembly [49], while GIP‐2 is a component of the γ‐tubulin ring complex needed for the nucleation of microtubules during mitotic spindle formation [50]. Faulty spindle organization is a common cause of embryonic lethality in *C. elegans* [58, 59] and dysregulation of this process could explain the observed embryonic lethality in *pph‐7* mutants. However, a potential role of PPH‐7 in spindle organization would need to be confirmed in further experiments.

Since PPH‐7 encodes a phospho‐tyrosine phosphatase, we also performed phosphoproteomics. Most of the changes that we detected between wild‐type and mutants, however, were in the levels of phosphoserine and ‐threonine. We assume that PPH‐7 may directly or indirectly affect serine/threonine kinases. This caveat complicated the analysis. Nevertheless, when we concentrated on difference in phospho‐tyrosines, a few changes were observed. We detected 11 proteins that were more phosphorylated in the mutant animals compared to wild type. Among others, these included genes involved in development: DLG‐1, the *Drosophila* disc large homolog, which is essential for embryonic development in *C. elegans* [60], VIT‐2, a vitellogenin acting as an energy source during development [61] and SPAT‐2, a protein of unknown function but which likely is involved in asymmetric cell division in the early embryo [62]. It is tempting to speculate that the altered phosphorylation state of these proteins contributes to the embryonic lethal phenotype, we observed in *pph‐7* mutants. Furthermore, we detected PAT‐2 and PAT‐12, which have been linked to ECM function, which in turn is also controlled by VHL‐1 activity [21, 24]. Thus, it is conceivable that PPH‐7 acts in a pathway that also requires VHL‐1 function. Further experiments are needed to establish the precise pathway and its function.

We identified a novel protein tyrosine phosphatase of the PTPN family in *C. elegans*, required for fertility and embryonic development at elevated temperatures and which may play a role in the regulation of von Hippel–Lindau targets.

## Conflict of interest

The authors declare no conflict of interest.

### Peer review

The peer review history for this article is available at https://www.webofscience.com/api/gateway/wos/peer‐review/10.1002/2211‐5463.13771.

## Author contributions

CAF and ASpa conceived the project. CAF, DR, and NCK acquired data. CAF, DR, NCK, JAS, ASch, and ASpa analyzed and interpreted the data. CAF and ASpa wrote the manuscript.

## Supporting information


**Fig. S1.** (A) SNP mapping of the mutation in the FA50 mutant. Map ratios are shown for 3 SNPs on each *C. elegans* chromosome. A low map ration indicates that the corresponding SNP is in proximity of the mutation. The three bars for every chromosome indicate a SNP localizing to the beginning, the middle and the end of the chromosome, respectively (exact position of SNPs is shown in Table S1). Chromosome I and IV exhibit the lowest average map ratios as well as the lowest map ratios for single SNPs. (B) Timing of egg laying at 26°C. Number of eggs laid per worm at 26°C was determined very day for *n* = 10 worms per experiment for *N* = 3 biological replicates. Data are presented as mean +/− SD. Statistical significance was determined using two‐way ANOVA: **** p ≤ 0.0001. (C) Microinjection of Y71F9AL.4 plasmids with or without fluorescent tags in mutant or wild‐type animals. Transformed F1 worms were identified by their red pharynx (co‐injection marker). Presence of the red pharynx in F2 worms would indicate heritable expression (stable transgenic lines). For injection of a non‐toxic construct, the expected amount of transformed worms would be ~25 times higher than observed and ~ 10% of transformed worms would result in stable transgenic lines [65].

**Table S1.** SNPs used for SNP mapping. Name and position of every SNP used for the SNP mapping. 3 SNPs, which map to both arms as well as the central region of the chromosome, were analyzed for each of the 6 chromosomes.

**Table S2.** PPH‐7 modulates the levels spermatogenesis proteins and *vhl‐1* targets. Fold change and q‐value for all proteins that are significantly regulated in *af5* and/or *tm5332* compared to wild type. Arrows indicate up‐ (▲) or downregulation (▼) in the *pph‐7* mutants.

**Table S3.** S/T‐Phosphatases/‐Kinases and components of the ubiquitin degradation machinery are more phosphorylated in *pph‐7* mutants. Fold change and p‐value for selected phospho‐peptides enriched in *af5* and *tm5332* compared to wild type. All listed phospho‐peptides map to Serine/Threonine kinases and phosphatases or components of the ubiquitin degradation machinery and exhibit at least a 4‐fold change in both *pph‐7* mutant alleles. Arrows indicate increased (▲) or decreased (▼) phosphorylation in the *pph‐7* mutants.

**Table S4.** Putative PPH‐7 targets include ECM components. Fold change and p‐value for phospho‐peptides containing a phospho‐tyrosine. Only putative PPH‐7 targets that are enriched at least 4‐fold in both *pph‐7* mutants, *af5* and *tm5332*, compared to wild type are shown. Arrows indicate increased (▲) or decreased (▼) phosphorylation in the *pph‐7* mutants.

## Data Availability

Most data are contained in the manuscript. Raw proteome/phosphoproteome data and result files are available at the MassIVE repository (https://massive.ucsd.edu), Identifier MSV000092663.
