# Supplementary figures and images for: The protein tyrosine phosphatase PPH‐7 is required for fertility and embryonic development in C. elegans at elevated temperatures
Source: FEBS Open Bio. 2024 Feb 6;14(3):390–409. doi: 10.1002/2211-5463.13771 (PMC10909979; doi:10.1002/2211-5463.13771)

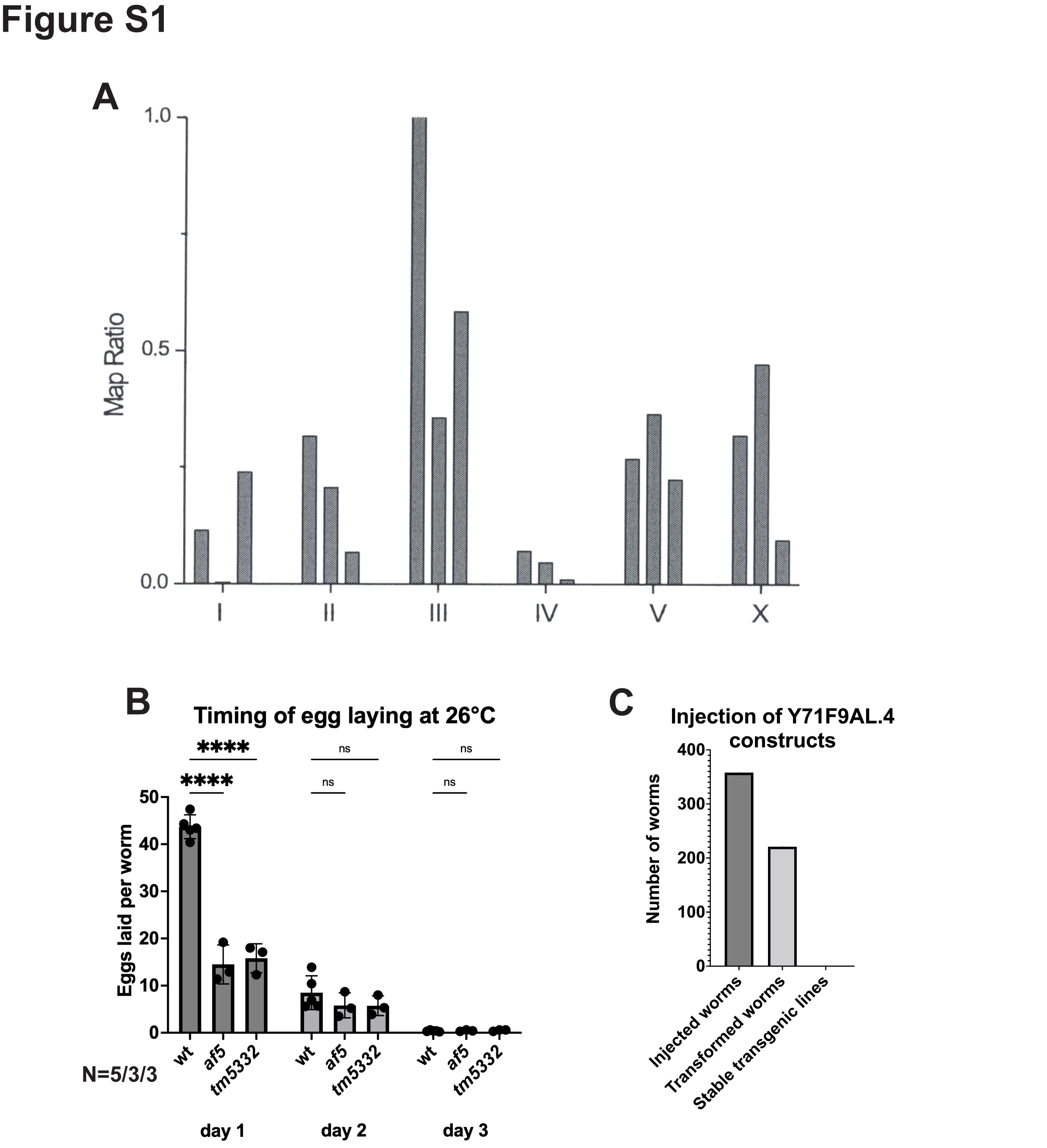

Supplement: Supplementary file 1 — Fig. S1. (A) SNP mapping of the mutation in the FA50 mutant. Map ratios are shown for 3 SNPs on each C. elegans chromosome. A low map ration indicates that the corresponding SNP is in proximity of the mutation. The three bars for every chromosome indicate a SNP localizing to the beginning, the middle and the end of the chromosome, respectively (exact position of SNPs is shown in Table S1). Chromosome I and IV exhibit the lowest average map ratios as well as the lowest map ratios for single SNPs. (B) Timing of egg laying at 26°C. Number of eggs laid per worm at 26°C was determined very day for n = 10 worms per experiment for N = 3 biological replicates. Data are presented as mean +/− SD. Statistical significance was determined using two‐way ANOVA: **** p ≤ 0.0001. (C) Microinjection of Y71F9AL.4 plasmids with or without fluorescent tags in mutant or wild‐type animals. Transformed F1 worms were identified by their red pharynx (co‐injection marker). Presence of the red pharynx in F2 worms would indicate heritable expression (stable transgenic lines). For injection of a non‐toxic construct, the expected amount of transformed worms would be ~25 times higher than observed and ~ 10% of transformed worms would result in stable transgenic lines [65]. [file FEB4-14-390-s004.tiff]
