# Supplementary material for: The protein tyrosine phosphatase PPH‐7 is required for fertility and embryonic development in C. elegans at elevated temperatures
Source: FEBS Open Bio. 2024 Feb 6;14(3):390–409. doi: 10.1002/2211-5463.13771 (PMC10909979; doi:10.1002/2211-5463.13771)
Supplement: Supplementary file 2 — Table S1. SNPs used for SNP mapping. Name and position of every SNP used for the SNP mapping. 3 SNPs, which map to both arms as well as the central region of the chromosome, were analyzed for each of the 6 chromosomes. [file FEB4-14-390-s002.pdf]

**SNPs used for SNP mapping**

| Chromosome | SNP Allel | Cosmid | Genomic positio | Genomic position of cosmid |
|---|---|---|---|---|
| I | **pkP1051** | ZC123 | -21.5 | I:803399..847690 |
| | **pKP1057** | K04F10 | 0.9 | I:6332186..6367598 |
| | **pKP1071** | C37A5 | 23.1 | I:14137461..14180111 |
| II | **pKP2101** | T01D1 | -16.2 | II:162669..194551 |
| | **pKP2107** | T13C2 | 0.1 | II:6766969..6799260 |
| | **pKP2117** | Y48E1B | 15.9 | II:13527912..13528351 |
| III | **pKP3045** | K02F3 | -26.1 | III:822701..860086 |
| | **pKP3049** | F10E9 | -0.3 | III:8278844..8326077 |
| | **pKP3075** | Y75B8A | 15.8 | III:12069105..12367511 |
| IV | **pKP4049** | Y66H1A | -24.8 | IV:425890..453915 |
| | **pKP4034** | R105 | 0.7 | IV:4618783..4646067 |
| | **pKP4045** | M199 | 18.1 | IV:15101279..15140461 |
| V | **pKP5083** | W03F9 | -19.3 | V:124309..167186 |
| | **pKP5062** | F20D6 | 1 | V:8158797..8199249 |
| | **pKP5082** | Y51A2D | 19.3 | V:18479147..18618405 |
| X | **pKP6100** | F28C10 | -19.5 | X:530603..545669 |
| | **pKP6110** | F45E1 | -0.8 | X:7972785..8010906 |
| | **pKP6124** | F23D12 | 17.3 | X:14413673..14453779 |
