# Supplementary material for: The protein tyrosine phosphatase PPH‐7 is required for fertility and embryonic development in C. elegans at elevated temperatures
Source: FEBS Open Bio. 2024 Feb 6;14(3):390–409. doi: 10.1002/2211-5463.13771 (PMC10909979; doi:10.1002/2211-5463.13771)
Supplement: Supplementary file 3 — Table S2. PPH‐7 modulates the levels spermatogenesis proteins and vhl‐1 targets. Fold change and q‐value for all proteins that are significantly regulated in af5 and/or tm5332 compared to wild type. Arrows indicate up‐ (▲) or downregulation (▼) in the pph‐7 mutants. [file FEB4-14-390-s005.pdf]

PPH-7 modulates the levels spermatogenesis proteins and vhl-1 targets

Group	Protein	Uniprot accession	SA50-7 vs N2		tm5332 vs N2	
			fold change	q-value	fold change	q-value
Supressed by VHL-1 (•)	MCT-2	Q9UAT3	▼ 3.41176836685593	0.007538986	▼ 3.32775902018549	0.008504951
	CLEC-209	G5EBG4	▼ 4.95418693856522	0.009269684	▼ 3.26449856394631	0.029079618
	C01B4.6	G5ECR6	▼ 3.67656832253477	0.01531505	▼ 3.2405917449008	0.024138866
Spermatogenesis (•)	SMZ-1	Q18167	▼ 1.33609542612802	0.00274569	▼ 1.39957073670345	0.002274204
	SSQ-1	Q21294	▼ 1.35265838439082	0.028535018	▼ 1.44778249761728	0.01265734
	MSP-3	Q19832	▼ 1.36104570028293	0.045484676	▼ 1.50661643977774	0.01265734
Spindle organization (•)	TPXL-1	G5EDE7	▲ 1.35197166344433	0.009137289	▲ 1.39249571989838	0.008504951
	GIP-2	G5EF84	▼ 1.69240136049014	0.011077041	▼ 1.44802556943661	0.04177504
	CYP-35b2	O44651	▼ 1.96569579449286	0.037706368	▼ 1.88542484886375	0.04177504
	F13E6.1	P55326	▲ 3.58561597776173	0.000686982	▲ 1.13646413971287	0.578164629
	ZK105.1	Q965Z4	▲ 1.59519638143369	0.09028991	▲ 2.17670980318983	0.01265734
	E04F6.9	Q19064	▲ 1.19375470346843	0.139290891	▲ 1.42486384344584	0.01265734
	F41B4.1	Q966J6	▲ 1.05679440328785	0.640015754	▲ 1.20367070960942	0.029079618
	TTR-6	O17345	▲ 1.21801997787257	0.529143498	▲ 1.63708902780847	0.044943796

Significantly regulated *af5* vs wild type and *tm5332* vs wild type
Significantly regulated in *af5* vs wild type
Significantly regulated in *tm5332* vs wild type
