# Supplementary material for: The protein tyrosine phosphatase PPH‐7 is required for fertility and embryonic development in C. elegans at elevated temperatures
Source: FEBS Open Bio. 2024 Feb 6;14(3):390–409. doi: 10.1002/2211-5463.13771 (PMC10909979; doi:10.1002/2211-5463.13771)
Supplement: Supplementary file 4 — Table S3. S/T‐Phosphatases/‐Kinases and components of the ubiquitin degradation machinery are more phosphorylated in pph‐7 mutants. Fold change and p‐value for selected phospho‐peptides enriched in af5 and tm5332 compared to wild type. All listed phospho‐peptides map to Serine/Threonine kinases and phosphatases or components of the ubiquitin degradation machinery and exhibit at least a 4‐fold change in both pph‐7 mutant alleles. Arrows indicate increased (▲) or decreased (▼) phosphorylation in the pph‐7 mutants. [file FEB4-14-390-s001.pdf]

S/T-Phosphatases/-Kinases and components of the ubiquitin degradation machinery are more phosphorylated in pph-7 mutants

Group	Protein	Uniprot accession	Peptide	[coordinate in peptide] PTM	Phosphorylated residue(s)	*af5* vs wild type		*tm5332* vs wild type	
						fold change	p-value	fold change	p-value
S/T-Kinases/ -Phosphatases (•)	**GCK-4**	A0A4V0IKG6	SRPSLSPQLR	[6] Phospho (ST)	S537	▲ 4736.67	5.49E-08	▲ 253.52	5.366E-05
	**PAR-1**	A0A2C9C376	RSSLDNIMKDR	[3] Phospho (ST)	S510	▲ 123.69	0.0001546	▲ 4.53	0.0610631
	**PPM-1.G**	P49595-2	KENEDASAEVVIENAEDKEEEEGSPK	[24] Phospho (ST)	S219	▲ 90.94	0.0001377	▲ 13.15	0.02845
	**UNC-82**	D1MN51	HLSLEKSVSPQR	[7] Phospho (ST)|[9] Phospho (ST)	S1413,S1415	▲ 60.55	0.0018849	▲ 39.60	0.0241207
	**H18N23.2**	H2KYT5	YRGESFEEEEMTTR	[5] Phospho (ST)	S177	▲ 35.40	0.0012844	▲ 27.58	0.0487255
	**MIG-15**	Q23356-2	SREESMSPPPPAPPPR	[1] Phospho (ST)|[7] Phospho (ST)	S526,S532	▲ 33.03	0.0034502	▲ 23.04	0.0068822
	**SEL-5**	G5ECQ3	KDETINEEDSEIDEQR	[10] Phospho (ST)	S694	▲30.68	0.0005974	▲ 39.63	0.0007874
	**PPFR-4**	Q9N4E9	FGHNPQNAPQSSAPAGAEAQESEEEVDDDEAR	[22] Phospho (ST)	S293	▲ 29.74	0.0004638	▲ 17.50	0.0079139
	**Y38H8A.4**	O62424	VNEESTDASENQEEPDELSPR	[19] Phospho (ST)	S20	▲ 23.09	0.0000289	▲ 8.52	0.0799089
	**MADD-3**	A0A3B1DR12	DEREDSSIEFIK	[7] Phospho (ST)	S188	▲ 10.55	0.0000993	▲ 5.70	0.0558883
	**CDK-1**	P34556	IGEGTYGVVYKGK	[5] Phospho (ST)|[6] Phospho (Y)	T32,Y33	▲ 9.78	0.0055234	▲ 5.06	0.2922835
	**EGG-3**	Q20402	SDSIEFKDAVINEK	[3] Phospho (ST)	S18	▲ 9.032	0.0005111	▲ 4.15	0.0129378
	**MAK-2**	Q965G5	GGASPKDDPMDDIKEEEKDDEEEK	[4] Phospho (ST)	S343	▲ 8.51	0.0001723	▲ 4.10	0.0761562
	**PPFR-2**	P91198	SPINTSPSSSPK	[9] Phospho (ST)|[10] Phospho (ST)	S304,S305	▲ 5.71	0.0007679	▲ 5.41	0.0006588
ubiquitination/ deubiquitination (•)	**UBR-5**	G5EDT9	RADSEENDEYGGENHSFSAVR	[4] Phospho (ST)	S1054	▲ 70.18	0.0662491	▲ 67.57	0.0099372
	**MATH-33**	N1NSD8	IDMFDSDDEAR	[6] Phospho (ST)	S614	▲ 12.89	0.0073942	▲ 7.57	0.0155408
	**UBXN-2**	U4PBA6	DIGNNDDGGPDSDDSGADAAER	[12] Phospho (ST)	S21	▲ 10.17	0.0013691	▲ 8.72	0.0032393
