# Supplementary material for: The protein tyrosine phosphatase PPH‐7 is required for fertility and embryonic development in C. elegans at elevated temperatures
Source: FEBS Open Bio. 2024 Feb 6;14(3):390–409. doi: 10.1002/2211-5463.13771 (PMC10909979; doi:10.1002/2211-5463.13771)
Supplement: Supplementary file 5 — Table S4. Putative PPH‐7 targets include ECM components. Fold change and p‐value for phospho‐peptides containing a phospho‐tyrosine. Only putative PPH‐7 targets that are enriched at least 4‐fold in both pph‐7 mutants, af5 and tm5332, compared to wild type are shown. Arrows indicate increased (▲) or decreased (▼) phosphorylation in the pph‐7 mutants. [file FEB4-14-390-s003.pdf]

Putative PPH-7 targets include ECM components

Group	Protein	Uniprot accession	Peptide	[coordinate in peptide] PTM	Phosphorylated residue(s)	*af5* vs wild type		*tm5332* vs wild type	
						fold change	p-value	fold change	p-value
Putative PPH-7 targets (•)	**DLG-1**	G5ECY0	AMSGEEAQAQYSR	[11] Phospho (Y)	Y919	▲ 669.53	0.018184319	▲ 20.39	0.574928951
	**PAT-2**	P34446	TPNEYSREEDDESYEDETTTQSQSTR	[1] Phospho (ST)|[14] Phospho (Y)	T914,Y927	▲ 152.95	0.006388144	▲ 5.57	0.383408643
	**PAT-12**	Q95QA6-3	TEQLPGGYETFNKER	[8] Phospho (Y)	Y617	▲ 141.58	0.000373943	▲ 11.94	0.049581424
	**H11E01.3**	A0A5E4LWF5A3:C14	IDLEAYHNSPTKESTSPVTVNPVEVR	[6] Phospho (Y)|[11] Phospho (ST)	Y1193,T1198	▲ 81.69	0.002156264	▲ 7.65	0.037181732
	**VIT-2**	P05690	KYERDEEQSDEYSSEETYDYEQENTKK	[12] Phospho (Y)|[13] Phospho (ST)	Y1502,S1503	▲ 36.27	0.00956798	▲ 51.45	0.006907227
	**SPAT-2**	A0A1I6CM87	RSSSGAAQQSEEDDDQSYVSAQENLGGSNSR	[18] Phospho (Y)	Y584	▲ 18.95	0.000661922	▲ 8.99	0.037938029
	**SPAT-2**	A0A1I6CM87	SSSGAAQQSEEDDDQSYVSAQENLGGSNSR	[17] Phospho (Y)	Y584	▲ 18.51	0.000160673	▲ 6.89	0.043298032
	**T20B5.2**	Q22600	HKLQYDEVSPR	[5] Phospho (Y)	Y125	▲ 14.7	0.048292825	▲ 6.99	0.097421622
	**IFC-2**	Q21067-2	THSSSNTSYSNVPASR	[4] Phospho (ST)|[5] Phospho (ST)|[9] Phospho (Y)	433S, 434S, 438Y	▲ 14.33	0.080751326	▲ 13.10	0.092507243
	**CDK-1**	P34556	IGEGTYGVVYKGK	[5] Phospho (ST)|[6] Phospho (Y)	T32,Y33	▲ 9.78	0.005523371	▲ 5.06	0.292283491
	**UNC-70**	E0AHA7	YLYDTLR	[3] Phospho (Y)	Y820	▲ 8.93	0.527006789	▲ 4.25	0.902770202
	**NEX-1**	G5EEA8	TSPYATIVDAR	[4] Phospho (Y)	Y5	▲ 8.08	0.013523163	▲ 4.3	0.033975069
